# Incidence and clinical progression of asymptomatic peripherally inserted central catheter*–*related thrombosis in solid neoplasm patients: ultrasound insights from a prospective cohort study

**DOI:** 10.1016/j.rpth.2024.102391

**Published:** 2024-03-27

**Authors:** Mattia Cominacini, Sergio De Marchi, Federica Tosi, Elia Piccinno, Alessandro Dal Corso, Elisa Dalla Grana, Francesca Stefani, Luca Dalle Carbonare

**Affiliations:** 1 Department of Engineering for Innovative Medicine, University of Verona, Verona, Italy; 2 Department of Angiology, Integrated University Hospital of Verona, Verona, Italy; 3 Department of Emergency Medicine, Integrated University Hospital of Verona, Verona, Italy

**Keywords:** central venous catheter thrombosis, Doppler, neoplasms, PICC line catheterization, secondary, ultrasound imaging, upper exremitiy deep vein thrombosis, vascular access devices

## Abstract

**Background:**

Managing central venous catheters in patients with neoplasms is challenging, and peripherally inserted central catheter PORT (PICC-PORT) has emerged as a promising option for safety and efficacy. However, understanding the clinical progression of catheter-related thrombosis (CRT) in cancer patients with central venous catheters remains limited, especially in certain neoplasm types associated with a higher risk of venous thrombosis.

**Objectives:**

This study aims to assess the effectiveness of ultrasound-guided management in detecting and treating asymptomatic CRT in cancer patients with PICC.

**Methods:**

In this prospective cohort study of 120 patients with solid neoplasms receiving chemotherapy, we investigated the incidence of isolated upper-extremity superficial vein thrombosis, upper-extremity deep vein thrombosis, and fibrin sheath formation through ultrasound follow-up at 30 and 90 days after catheter insertion. We analyzed risk factors associated with CRT and compared incidence rates between PICC-PORT and traditional PICC.

**Results:**

Among the cohort, 69 patients (57.5%) had high-risk thromboembolic neoplasm, and 31 cases (25.8%) of CRT were observed, mostly within 30 days, with only 7 cases (22.6%) showing symptoms. Traditional PICC use (odds ratio, 5.86; 95% CI, 1.14-30) and high-risk thromboembolic neoplasm (odds ratio, 4.46; 95% CI, 1.26-15.81) were identified as independent risk factors for CRT.

**Conclusion:**

The majority of CRT present asymptomatically within the first 30 days of venous catheter insertion in patients with solid neoplasms. Ultrasound follow-up is valuable for detecting asymptomatic CRT. The risk of CRT was lower with PICC-PORT than with PICC. Additionally, the risk of CRT was found to be higher in patients with high-risk thromboembolic neoplasms. It is crucial for larger studies to confirm the utility of treating asymptomatic thromboses and isolated superficial thrombosis.

## Essentials

• Peripherally inserted central catheters in cancer patients are safer and cost effective.
• Catheter-related thrombosis (CRT) disrupts treatment, but we still know little about it.
• The majority of CRT in cancer patients occurs asymptomatically, and ultrasound can identify them.
• Research is needed to define role of asymptomatic CRT and superficial vein thrombosis in oncology.

## Graphical abstract

**Figure:**
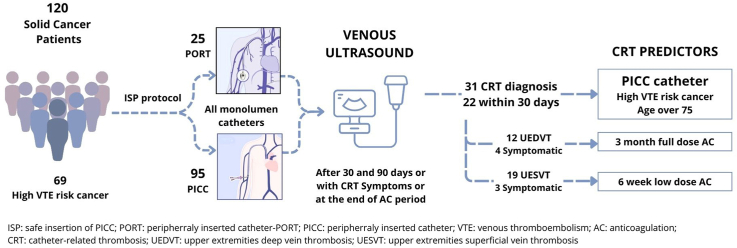

## 1 Introduction

Cancer significantly increases thrombosis risk, constituting 20% of all thrombotic events. In ambulatory cancer patients undergoing systemic chemotherapy, cancer-related thrombosis (CAT) is a leading cause of non–cancer-related mortality, especially in high-risk malignancies such as pancreatic, stomach, lung, ovarian, urothelial, and liver cancers [[1], [2], [3], [4], [5], [6]]. Access to a central vein for chemotherapy and blood collection is paramount in modern oncology. Peripherally inserted central catheters (PICCs) are preferred over central venous catheters (CVCs) in cancer patients due to lower cost, perceived safety, and ease of insertion outside the operating theater.

Cancer patients face a higher catheter-related thrombosis (CRT) risk compared with noncancer patients, with PICC associated with a higher incidence than CVC [[7], [8], [9]]. CRT can disrupt oncology treatment by causing venous access occlusion, leading to chemotherapy delays or suspension and potential severe complications [[8], [9], [10]]. Various scoring systems exist to assess thrombosis risk in cancer patients and CRT in patients with PICC [11,12]. While the Khorana risk score assesses thrombosis risk in cancer patients, it lacks validation for CRT. Similarly, the Michigan risk score, used for PICC-related thrombosis, lacks validation for cancer patients. Currently, no validated score evaluates the risk of PICC-related thrombosis in solid cancer patients. To address concerns about CRT with PICC, the PICC-PORT technique combines advanced insertion methods with proper catheter tip positioning. This technique has shown promising results in breast cancer patients and those with extensive burns, gaining popularity in Italian clinical centers as a primary choice for central vascular access devices due to perceived safety, efficacy, ease of insertion, and patient tolerance [[13], [14], [15], [16], [17]].

CRT is classified as upper-extremity superficial vein thrombosis (UESVT) or upper-extremity deep vein thrombosis (UEDVT) based on the affected venous vessel [18]. UEDVT primarily involves the subclavian vein and the axillary vein, with potential extension to the brachiocephalic trunk, superior vena cava, or internal jugular vein. UESVT mainly affects the basilic vein, humeral vein, and cephalic vein. UESVT, historically viewed as benign, occurs in 15% to 32% of hospitalized patients with intravenous catheters [19]. Cancer and the use of central catheters have been suggested as potential risk factors for the progression from UESVT to UEDVT [19]. Superficial vein thrombosis (SVT), once perceived as less severe than deep vein thrombosis or pulmonary embolism, is now recognized as interconnected with them. A recent study indicates an 8.7% cancer prevalence in SVT patients [11]. UEDVT is linked to complications such as recurrent thrombosis (8% at 5 years), pulmonary embolism (5%), superior vena cava syndrome, and postthrombotic venous insufficiency (20% untreated) [20,21]. Over 50% of cancer patients experience asymptomatic catheter-related UESVT and UEDVT [[22], [23], [24]]. The reasons for variations in symptoms among individuals with similar thrombi locations remain largely unknown [[22], [23], [24]].

CRT is often linked to the development of a fibrin sheath around the catheter tip [[25], [26], [27]]. The fibrin sheath, a layer of fibrin on the catheter’s surface, serves as a potential site for thrombus formation, though its exact role in CRT development is unclear. Notably, fibrin sheath formation does not involve the vessel wall and typically occurs within the first month after catheter placement. It can obstruct blood withdrawal, allowing infusion but impeding blood withdrawal [[25], [26], [27]].

The diagnosis of CRT and fibrin sheath is commonly performed using ultrasound, which is preferred for its noninvasive nature, lack of radiation exposure, and ease of use. Ultrasound with Color/Doppler techniques has demonstrated high sensitivity and specificity in detecting upper-limb venous thrombosis, currently, it is widely used as the first choice in clinical practice [[27], [28], [29]].

Understanding the etiology, complications, and ultrasound-guided management of these thrombotic events is critical for improving patient outcomes. This study aims to assess the effectiveness of ultrasound-guided management in detecting and treating asymptomatic CRT in cancer patients with PICC.

## 2 Methods

### 2.1 Study design

This retrospective analysis utilized prospectively collected data from a hospital database. The study included consecutive patients with cancer who underwent their first placement of a PICC or PICC-PORT between January 2020 and December 2022 in Azienda Ospedaliera Universitaria Integrata of Verona.

Inclusion criteria consisted of patients with any solid neoplasm requiring initiation of chemotherapy, need for the placement of a PICC or PICC-PORT, obtaining informed consent for study participation, or age over 18 years.

Patients were excluded if they had a communication disorder, patients already on anticoagulation therapy, hematological malignancy, severe renal failure or hepatic insufficiency (glomerular filtration rate <30 mL/min and Child-Pugh C), active major bleeding, severe thrombocytopenia (platelet count <50,000/mmc), pregnancy, hypersensitivity to anticoagulant medications, expected survival of <3 months or surgery in the previous 3 months.

Criteria for study withdrawal were withdrawal of informed consent, loss to follow-up, or removal of the catheter before the second ultrasound control.

The main goals of this study were to assess the occurrence of fibrin sheath and CRT, encompassing both superficial and deep cases, and considering symptomatic as well as asymptomatic instances. We classified UESVT as isolated superficial thrombosis, while UEDVT was considered as such whether it occurred independently or in association with UESVT. Secondary objectives included the examination of risk factors associated with CRT.

### 2.2 Samples and ethical considerations

Patient characteristics and laboratory data were collected from the electronic medical records system of our hospital. Written informed consent was obtained from all patients prior to the placement of the PICC or PICC-PORT. The study was registered in the Clinical Trial Registry (Clinicaltrial.gov ID: NCT05966909). The study complied with the revised ethical guidelines of the Declaration of Helsinki.

### 2.3 Procedure details of vascular access placement and catheter management

Insertion technique followed the recommendations of the Italian Group for the Study of Long-Term Central Venous Access Devices (GAVeCeLT), specifically their safe implantation of PICCs (ISP protocol), aiming to minimize associated risks of PICC placement [30]. Experienced nurses at our Venous Access Center performed all catheter implantations in a dedicated room, following local protocols and using antiseptic techniques and full protective personal sterile equipment. The catheters were inserted under local anesthesia. The choice of the catheter insertion site was guided by examination and preprocedural ultrasound (VIVID 7pro GE Medical System) to identify any local conditions that could contraindicate venous placement, such as previous venous thrombosis, axillary dissection, or soft tissue infection [14]. The choice of the vascular access site also took into account the catheter-to-vein ratio (≤0.33) to minimize intravascular trauma and the risk of thrombosis [31,32]. During the PICC insertion, venipuncture was guided by ultrasound, and the vein (basilic, brachial, cephalic) was cannulated at the third middle of the arm (green zone according to Dawson’s ZIM method) or at the proximal third of the arm (yellow zone of Dawson’s ZIM method) and then tunneled to the “green zone” [14]. A single brand of monolumen PICC (HealthPICC, Plan1Health) was used for all patients. The totally implanted peripherally inserted central venous access (PICC-PORT) was also inserted in the dedicated room by an experienced trained nurse. The catheter placement site was chosen, similar to traditional PICC, through examination and preprocedural ultrasound to identify an adequate vein diameter. Ultrasound-guided venipuncture was performed at the proximal third of the arm, and then the reservoir was buried in a subcutaneous pocket created in the middle third of the arm or near the site of venipuncture. The implanted device consisted of a 5Fr polyurethane catheter with a full titanium reservoir (HealtPort MiniMax, Plan1Health). The catheter tip location near the cavo-atrial junction for both PICC and PICC-PORT was always verified using intracavitary ECG (preferred method) or by chest X-ray. Maintenance of the implanted device was performed according to local institution protocols. Flushing and locking procedures were carried out with normal saline in a pulsatile manner before and after any infusion or at 2-month intervals if the catheter was not in use. In this study, the selection between PICC and PORT was primarily guided by the availability of supplies. However, in instances where both options were accessible, patient preference, particularly in terms of esthetic considerations, was considered whenever feasible. In PICC-PORT management, the reservoir was accessed using dedicated needles.

### 2.4 Ultrasound examination and follow-up

PICC placement guide and follow-up were performed by ultrasound using the VIVID 7 pro (GE Medical Systems—high definition linear probe). After PICC placement, all patients had follow-up ultrasound at 1 month and 3 months. If the patient had clinical symptoms related to upper-extremity thrombosis, ultrasound could be carried out at any time and, in case of CRT diagnosis, it was repeated at the end of the planned anticoagulation period.

The examination was done by a physician with over 20 years of experience in vascular ultrasound. The diagnostic maneuver for thrombosis detection was venous compression (CUS) produced by means of ultrasound probe starting from catheter insertion site up to basilic vein, brachial and subclavian-axillary segments, the examination was also extended to interior jugular and distal brachiocephalic veins. We did not perform ultrasound examinations on the limb contralateral to the catheter or the lower limbs unless specific symptoms suggestive of a thromboembolic event were present. SVT was established if thrombosis involved a superficial vein of the upper extremity, including the basilic and cephalic veins. DVT was established if a deep vein, such as the brachial vein, axillary vein, or subclavian vein, were affected. Fibrin sheath was defined as a hyperechogenic tissue around the catheter with ≤3 mm thickness , at least 20-mm long, with no relationship with the vein wall [27].

### 2.5 Statistical analyses

Statistical analyses were undertaken using JAMOVI [33,34]. Continuous variables (mean ± SE) were analyzed with a two-tailed Student’s *t*-test. Variables with a nonnormal distribution were analyzed using the Mann–Whitney U-test. Categorical variables were compared using the chi-squared test and Fisher’s exact test. The predictors of CRT were identified through binary logistic regression analysis following both univariate and multivariate analyses. The variables selected for the multivariate logistic regression model included those that approached statistical significance in the univariate analysis, along with some variables suggested in the literature [11]. *P* < .05 was considered significant.

## 3 Results

### 3.1 Patients

Between January 2020 and December 2022, 120 solid cancer patients receiving systemic chemotherapy and with a venous catheter (PICC or PICC-PORT) were enrolled in the study. The patients had a median age of 68 years (range: 32-86 years), with 58.3% (70 out of 120) being male. Table 1 provides the patient demographics and blood test results. Of the 120 patients, 51 (42.5%) had pancreatic cancer, 14 (11.7%) had breast cancer, 11 (9.2%) had cholangiocarcinoma, and the remaining patients had various other types of cancer, as shown in Table 1. A majority of the patients (57.5%) had high-risk thromboembolic cancer (stomach, pancreas, lung, ovarian, urothelial, or hepatocellular carcinoma). Table 1 displays the distribution of patients based on the Khorana score. Of the patients, 46.8% (51 out of 120) had a platelet count of more than 350.000/mmc and 5.6% (6 out of 120) had a white blood cell count of more than 11.000/mmc. No patient had a BMI of >35 kg/m^2^.

**Table 1:** Anthropometric and clinical characteristics of the study population.

Patient Characteristics^a^	Mean (SD)
Age (y)	66.17 (11.23)
Weight (Kg)	70.10 (14.07)
Laboratory values	Mean (SD)
Hb (g/dL)	12.42 (1.54)
Plts (mm^3^)	267,064 (120,496)
WBC (mm^3^)	6,815 (2,732)
eGFR (mL/min)	90.65 (29.8)
PT (INR)	1.06 (0.41)
aPTT (INR)	1.03 (0.14)
Fibrinogen (g/L)	1.92 (1.39)
Cancer type	Counts (% of total)
Pancreas	51 (42.5)
Breast	14 (11.7)
Biliary	13 (10.8)
Colorectal	11 (9.2)
Lung	7 (5.8)
Connective tissue	7 (5.8)
Stomach	5 (4.2)
HCC	4 (3.3)
Other	8 (6.7)
Catheter type	Counts (% of total)
PICC	95 (79.2)
Right side	73 (60.8)
Left side	22 (18.3)
PORT	25 (20.8)
Right side	23 (19.2)
Left side	2 (1.6)
Khorana score	Counts (% of total)
0	31 (25.8)
1	20 (16.7)
2	42 (35.0)
3	25 (20.8)
4	2 (1.7)

aPTT, activated partial thromboplastin time; GFR, estimated glomerular filtration rate (with Cockcroft-Gault); Hb, hemoglobin; HCC, hepatocellular carcinoma; Ht, hematocrit; MCV, mean cell volume; PICC, peripherally inserted central catheter; Plts, platelets; PORT, peripherally inserted central catheter PORT; PT, prothrombin time; WBC, white blood cell.

a All the patients who participated in the study were of Caucasian origin

### 3.2 Study flow

In 96 patients (80%), the catheter was implanted in the right arm and the basilic vein was the most used (88 were in the right basilic vein while 22 were in the left basilic vein). Table 1 shows the distribution of which vein was chosen for the placement of the venous catheter. In terms of the type of catheter used, 95 (79.2%) patients had PICC inserted, while 25 (20.8%) had PICC-PORT. All PICC-PORT were inserted into the basilic vein: 22 on the right and 3 on the left. The demographic and biochemical characteristics, as well as the type of cancer, were homogeneous between the PICC and PICC-PORT patient groups (data not shown).

### 3.3 Main outcomes

One CRT event occurred in 31 patients (25.8%), involving 12 cases of UEDVT, of which 5 were associated with UESVT, and 19 cases of isolated UESVT. All 12 cases of UEDVT were associated with PICC. Symptoms of CRT were reported by only 7 patients (22.6%), all of whom belonged to the PICC group. Among these symptomatic cases, 3 were identified as UEDVT associated with UESVT. Table 2 shows the distribution of CRT events and fibrin sheath encountered. Out of the 31 CRT detected, 22 were diagnosed 30 days after catheter insertion during the first ultrasound examination. Among these, 9 were UEDVT, of which 4 were associated with UESVT, and 13 were isolated UESVT.

**Table 2:** Chateter-related thrombosis observed during the study period.

Outcomes	PICC (*n* = 95)	PORT (*n* = 25)	Event location
All CRT	29	2	
All CRT Symptomatic	7	0	
All CRT Asymptomatic	22	2	
All UEDVT	12	0	5 RSV, 3 RSV+RAV, 2 RAV, 1 LAV, 1 LAV+LSV+LJV
UEDVT Symptomatic	4	0	
UEDVT Asymptomatic	8	0	
All UESVT	17	2	12 RBV, 7 LBV
UESVT Symptomatic	3	0	
UESVT Asymptomatic	14	2	
ALL FS	11	7	15 RBV, 1 LBV, 1 RSV

CRT, catheter-related thrombosis; FS, fibrin sheath; LAV, left axillary vein; LBV, left brachial vein; LJV, left jugular vein; LSV, left subclavian vein; PICC, peripherally inserted central catheter; PORT, peripherally inserted central catheter PORT; RAV, right axillary vein; RBV, right brachial vein; RSV, right subclavian vein; UEDVT, upper extremities deep vein thrombosis; UESVT, upper extremities superficial vein thrombosis,.

### 3.4 Predictors

Age, the use of PICC, and the presence of a high thrombotic risk cancer were found to be significant risk factors for CRT in a univariate logistic regression model (Table 3). However, platelet count, leukocyte count, and hemoglobin did not demonstrate statistical significance. In a multivariable regression model, considering the Khorana risk factors, the type of venous catheter used, age and gender, the only 2 predictors that retain their statistical significance are the presence of a PICC and the presence of a high-risk thrombosis neoplasm, with ORs of 5.86 (95% CI, 1.14-30) and 4.46 (95% CI, 1.26-15.81), respectively. Analysis of risk factors for UEDVT and isolated UESVT separately revealed no statistically significant differences. However, it is noteworthy that, despite the absence of statistical significance, the use of PICC appears to exhibit a potentially more thrombogenic trend compared with PICC-PORT (OR, 7.63; 95% CI, 0.44-133 for UEDVT; OR, 3.47; 95% CI, 0.76-15.9 for UESVT). The presence of a fibrin sheath at the initial ultrasound has not been demonstrated as a significant risk factor for subsequent CRT development (OR, 1.33; 95% CI, 0.39-4.53; *P* = .64). Out of the 12 patients diagnosed with UEDVT, 8 received treatment with fondaparinux and the remaining 4 were treated with direct oral anticoagulants (DOACs) such as rivaroxaban or edoxaban. Additionally, out of the 19 patients diagnosed with isolated UESVT, 10 were treated with fondaparinux, and 9 were treated with rivaroxaban. All cases of thromboembolisms resolved within the prescribed therapeutic time frame, which was 3 months for UEDVT and 6 weeks for UESVT. No significant bleeding occurred, and no catheter was removed during the follow-up period.

**Table 3:** Univariate analysis of thrombotic events.

Predictors^a^ (reference group)	OR	95%CI	Cases (*n*)	Noncases (*n*)
Device type (PORT)	5.05	1.12-22.86	29	66
Cancer type (not high thrombotic risk cancer)^b^	1.79	0.76-4.24	21	48
	OR (per 1 SD higher)			
Age	2.62	0.56-3.16	9	12
White blood cell	1.14	0.34-3.82	4	13
Platelets	0.83	0.24-2.88	4	10
	OR (per 1 SD lower)			
Hemoglobin	1.10	0.37-3.32	5	17

PICC, peripherally inserted central catheter; PORT, peripherally inserted central catheter PORT.

a unadjusted variables

b High Thrombotic Risk Cancer: pancreatic, stomach, lung, ovarian, urothelial and liver cancers

## 4 Discussion

In this study, we investigated the incidence and risk factors of CRT in solid cancer patients undergoing systemic chemotherapy with venous catheter, specifically PICC and PICC-PORT. The results revealed that the majority of thrombosis occurred asymptomatically, especially in UESVT, and within the first month after catheter insertion. It is important to note that there is a paucity of outcome data, and the literature lacks a comprehensive understanding of the risks and clinical implications associated with clots identified asymptomatically. This emphasizes the need for additional research to clarify the potential implications and risks associated with asymptomatic thromboses, which currently remain relatively understudied in the existing body of literature.

Additionally, the study cautiously investigated the use of PICC-PORT, uncovering promising outcomes when compared with traditional PICC. This potential improvement might be attributed to reduced trauma to the vein wall over time, potentially contributing to a lower incidence of thrombosis. There is still a lack of specific recommendations or selection criteria for PICC types in the current literature and the increasing popularity of PICC-PORT warrants further research and comparison with other types of venous access devices. A significant aspect of this study is the inclusion of patients with high-risk thromboembolic cancers, such as pancreatic, which is associated with an increased risk of thrombotic events. This highlights the relevance of investigating management of thrombosis in patients with specific cancer types prone to thromboembolism. Furthermore, the study population included a substantial number of elderly cancer patients who are increasingly receiving chemotherapy due to advancements in treatment options with reduced toxicity. Thus, ensuring safe venous access is crucial in this vulnerable population. The study’s findings suggest the value of ultrasound follow-up for early detection and management of asymptomatic CRT in cancer patients; nevertheless, there is limited evidence available to support the hypothesis that treating UESVT associated with PICC in cancer patients leads to improved outcomes [[35], [36], [37], [38]]. It is worth noting that the presence of a fibrin sheath around the catheter tip did not emerge as a significant risk factor for CRT. Additionally, hemoglobin, leukocyte count, and platelet count did not prove to be significant factors. This suggests that other factors may play a more substantial role in thrombus formation, requiring further investigation into the etiology and mechanisms of CRT.

While this study provides valuable insights, it is important to acknowledge its limitations. The retrospective analysis and reliance on prospectively collected data introduce selection biases. The study’s single-institution focus may limit the generalizability of findings. The relatively small sample size underscores the need for larger cohorts to validate results. Additionally, the study lacks randomization or standardized protocols for catheter and anticoagulant selection, and it did not consider chemotherapy type and disease stage, known thrombosis risk factors.

## 5 Conclusions

Despite current guidelines predominantly addressing the management of symptomatic thrombosis, our study reveals that these constitute only a minority of venous thrombotic events in solid cancer patients with a placed PICC. The use of ultrasound surveillance may be valuable for facilitating the timely initiation of interventions, potentially contributing to the prevention of thrombosis progression or embolic events. Our study revealed that the majority of thrombosis occurs within the initial 30 days after venous catheter placement. Compared with traditional PICCs, PICC-PORTs in our study demonstrate lower thrombogenicity. Moreover, our findings confirm high-risk thrombotic neoplasms as a significant risk factor for the development of CRT. Currently, there is insufficient solid evidence supporting antithrombotic prophylaxis in this context [39].

Further research is essential to establish definitive recommendations for antithrombotic prophylaxis or asymptomatic CRT treatment utility in this specific scenario. Ultimately, these findings contribute to the evolving understanding of venous thrombosis management in cancer patients, with the goal of improving patient outcomes and enhancing the overall quality of care in oncology practice.

## References

[1] Imberti D., Agnelli G., Ageno W., Moia M., Palareti G., Pistelli R. (2008). Clinical characteristics and management of cancer-associated acute venous thromboembolism: findings from the MASTER Registry. Haematologica.

[2] Chew H.K., Wun T., Harvey D., Zhou H., White R.H. (2006). Incidence of venous thromboembolism and its effect on survival among patients with common cancers. Arch Intern Med.

[3] Khorana A.A., Francis C.W., Culakova E., Kuderer N.M., Lyman G.H. (2007). Thromboembolism is a leading cause of death in cancer patients receiving outpatient chemotherapy. J Thromb Haemost.

[4] Khorana A.A., Fine R.L. (2004). Pancreatic cancer and thromboembolic disease. Lancet Oncol.

[5] Katholing A., Rietbrock S., Bamber L., Martinez C., Cohen A.T. (2017). Epidemiology of first and recurrent venous thromboembolism in patients with active cancer. Thromb Haemost.

[6] Connolly G.C., Chen R., Hyrien O., Mantry P., Bozorgzadeh A., Abt P. (2008). Incidence, risk factors and consequences of portal vein and systemic thromboses in hepatocellular carcinoma. Thromb Res.

[7] Marin A., Bull L., Kinzie M., Andresen M. (2021). Central catheter-associated deep vein thrombosis in cancer: clinical course, prophylaxis, treatment. BMJ Support Palliat Care.

[8] Patel G.S., Jain K., Kumar R., Strickland A.H., Pellegrini L., Slavotinek J. (2013). Comparison of peripherally inserted central venous catheters (PICC) versus subcutaneously implanted port-chamber catheters by complication and cost for patients receiving chemotherapy for non-haematological malignancies. Support Care Cancer.

[9] Pu Y.-L., Li Z.-S., Zhi X.-X., Shi Y.-A., Meng A.-F., Cheng F. (2019). Complications and costs of peripherally inserted central venous catheters compared with implantable port catheters for cancer patients. Cancer Nurs.

[10] Taxbro K., Hammarskjöld F., Thelin B., Lewin F., Hagman H., Hanberger H., Berg S. (2019). Clinical impact of peripherally inserted central catheters vs implanted port catheters in patients with cancer: an open-label, randomised, two-centre trial. Br J Anaesth.

[11] Khorana A.A., Kuderer N.M., Culakova E., Lyman G.H., Francis C.W. (2008). Development and validation of a predictive model for chemotherapy-associated thrombosis. Blood.

[12] Chopra V., Kaatz S., Conlon A., Paje D., Grant P.J., Rogers M.A.M. (2017). The Michigan Risk Score to predict peripherally inserted central catheter-associated thrombosis. J Thromb Haemost.

[13] Bertoglio S., Annetta M.G., Brescia F., Emoli A., Fabiani F., Fino M. (2022).

[14] Dawson R.B. (2011). PICC Zone Insertion MethodTM (ZIMTM): a systematic approach to determine the ideal insertion site for PICCs in the upper arm. J Assoc Vasc Access.

[15] Shen Y., Wang Q., Li X., Chang J., Zhou X. (2022). Transverse incision versus longitudinal incision in the implantation of PICC-port: A before-after study. J Vasc Access.

[16] Bertoglio S., Cafiero F., Meszaros P., Varaldo E., Blondeaux E., Molinelli C. (2019). PICC-PORT totally implantable vascular access device in breast cancer patients undergoing chemotherapy. J Vasc Access.

[17] Merlicco D., Lombardi M., Fino M.C. (2020). PICC-PORT: Valid indication to placement in patient with results of extensive skin burns of the neck and chest in oncology. The first case in the scientific literature. Int J Surg Case Rep.

[18] Ploton G., Pistorius M.-A., Raimbeau A., Denis Le Seve J., Bergère G., Ngohou C. (2020). A STROBE cohort study of 755 deep and superficial upper-extremity vein thrombosis. Medicine.

[19] Yuen H.L.A., Fang W., Tran H., Chunilal S.D. (2022). Cannula provoked upper extremity superficial vein thrombophlebitis: are we overtreating?. Int Med J.

[20] Bleker S.M., van Es N., Kleinjan A., Büller H.R., Kamphuisen P.W., Aggarwal A. (2016). Current management strategies and long-term clinical outcomes of upper extremity venous thrombosis. J Thromb Haemost.

[21] Joffe H.V., Kucher N., Tapson V.F., Goldhaber S.Z. (2004). Upper-extremity deep vein thrombosis. Circulation.

[22] Wang G., Li Y., Wu C., Guo L., Hao L., Liao H. (2020). The clinical features and related factors of PICC-related upper extremity asymptomatic venous thrombosis in cancer patients. Medicine.

[23] Chen P., Zhu B., Wan G., Qin L. (2021). The incidence of asymptomatic thrombosis related to peripherally inserted central catheter in adults: a systematic review and meta-analysis People’s. Nursing Open.

[24] Gao Y., Liu Y., Chen W., Wei L., Song L., Ma X. (2015). Peripherally inserted central catheter thrombosis incidence and risk factors in cancer patients: a double-center prospective investigation. Ther Clin Risk Manag.

[25] Passaro G., Pittiruti M., La Greca A. (2020). The fibroblastic sleeve, the neglected complication of venous access devices: a narrative review. J Vasc Access.

[26] Trezza C., Califano C., Iovino V., D’Ambrosio C., Grimaldi G., Pittiruti M. (2020). Incidence of fibroblastic sleeve and of catheter-related venous thrombosis in peripherally inserted central catheters: a prospective study on oncological and hematological patients. J Vasc Access.

[27] Boddi M., Villa G., Chiostri M., De Antoniis F., De Fanti I., Spinelli A. (2015). Incidence of ultrasound-detected asymptomatic long-term central vein catheter-related thrombosis and fibrin sheath in cancer patients. Eur J Haematol.

[28] Li X., Wang G., Yan K., Yin S., Wang H., Wang Y. (2021). The incidence, risk factors, and patterns of peripherally inserted central catheter-related venous thrombosis in cancer patients followed up by ultrasound. Cancer Manag Res.

[29] Mustafa B.O., Rathbun S.W., Whitsett T.L., Raskob G.E. (2002). Sensitivity and specificity of ultrasonography in the diagnosis of upper extremity deep vein thrombosis. Arch Intern Med.

[30] Emoli A., Cappuccio S., Marche B., Musarò A., Scoppettuolo G., Pittiruti M. (2014). Gruppo Aperto di Studio sugli Accessi Venosi Centrali a Lungo Termine. Il protocollo ‘ISP’ (Inserzione Sicura dei PICC): un “bundle” di otto raccomandazioni per minimizzare le complicanze legate all’impianto dei cateteri centrali ad inserimento periferico (PICC) [The ISP (Safe Insertion of PICCs) protocol: a bundle of 8 recommendations to minimize the complications related to the peripherally inserted central venous catheters (PICC)]. Assist Inferm Ric.

[31] Nifong T.P., McDevitt T.J. (2011). The effect of catheter to vein ratio on blood flow rates in a simulated model of peripherally inserted central venous catheters. Chest.

[32] Sharp R., Cummings M., Fielder A., Mikocka-Walus A., Grech C., Esterman A. (2015). The catheter to vein ratio and rates of symptomatic venous thromboembolism in patients with a peripherally inserted central catheter (PICC): a prospective cohort study. Int J Nurs Stud.

[33] The jamovi project (2022) jamovi. (Version 2.3) [Computer Software]. https://www.jamovi.org.

[34] R: A Language and environment for statistical computing. (Version 4.1) [Computer software]. https://cran.r-project.org.

[35] Debourdeau P., Bertoletti L., Font C., López-Núñez J.J., Gómez-Cuervo C., Mahe I. (2023). Three-month outcomes in cancer patients with superficial or deep vein thrombosis in the lower limbs: results from the RIETE Registry. Cancers (Basel).

[36] Langer F., Gerlach H.E., Schimke A., Heinken A., Hoffmann U., Noppeney T. (2022). Clinical outcomes of cancer-associated isolated superficial vein thrombosis in daily practice. Thromb Res.

[37] Hirmerová J., Seidlerová J., Šubrt I., Hajšmanová Z. (2022). Prevalence of cancer in patients with superficial vein thrombosis and its clinical importance. J Vasc Surg Venous Lymphat Disord.

[38] Galanaud J.P., Blaise S., Sevestre M.A., Terrisse H., Pernod G., Gaillard C. (2018). Long-term outcomes of isolated superficial vein thrombosis in patients with active cancer. Thromb Res.

[39] Key N.S., Khorana A.A., Kuderer N.M., Bohlke K., Lee A.Y.Y., Arcelus J.I. (2020). Venous thromboembolism prophylaxis and treatment in patients with cancer: ASCO clinical practice guideline update. J Clin Oncol.

